# Transdiagnostic association between subjective insomnia and depressive symptoms in major psychiatric disorders

**DOI:** 10.3389/fpsyt.2023.1114945

**Published:** 2023-04-24

**Authors:** Suguru Nakajima, Yoshiyuki Kaneko, Nobukuni Fujii, Jun Kizuki, Kaori Saitoh, Kentaro Nagao, Aoi Kawamura, Takuya Yoshiike, Hiroshi Kadotani, Naoto Yamada, Makoto Uchiyama, Kenichi Kuriyama, Masahiro Suzuki

**Affiliations:** ^1^Department of Psychiatry, Nihon University School of Medicine, Tokyo, Japan; ^2^Department of Sleep-Wake Disorders, National Center of Neurology and Psychiatry, National Institute of Mental Health, Tokyo, Japan; ^3^Department of Psychiatry, Shiga University of Medical Science, Shiga, Japan; ^4^Kamibayashi Memorial Hospital, Aichi, Japan; ^5^Tokyo Adachi Hospital, Tokyo, Japan

**Keywords:** insomnia, depressive symptoms, sleep, sleep electroencephalography (EEG), psychiatric disorders

## Abstract

In psychiatric disorders, comorbid depressive symptoms are associated with clinically important issues such as reduced quality of life, a poor prognosis, and increased suicide risk. Previous studies have found a close relationship between insomnia and depressive symptoms in major depressive disorder (MDD), and that actively improving insomnia heightens the improvement of depressive symptoms. This study aimed to investigate whether the association between insomnia and depressive symptoms is also found in other psychiatric disorders besides MDD. The subjects were 144 patients with MDD (*n* = 71), schizophrenia (*n* = 25), bipolar disorder (*n* = 22), or anxiety disorders (*n* = 26). Sleep status was assessed subjectively and objectively using the Athens Insomnia Scale (AIS) and sleep electroencephalography (EEG), respectively. Sleep EEG was performed using a portable EEG device. Depressive symptoms were assessed using the Beck Depression Inventory. Subjective insomnia, as defined by the AIS, was associated with depressive symptoms in all disorders. Moreover, in schizophrenia, a relation between depressive symptoms and insomnia was also found by objective sleep assessment methods using sleep EEG. Our findings suggest that the association between subjective insomnia and depressive symptoms is a transdiagnostic feature in major psychiatric disorders. Further studies are needed to clarify whether therapeutic interventions for comorbid insomnia can improve depressive symptoms in major psychiatric disorders, similar to MDD.

## 1. Introduction

Depressive symptoms, such as depressed mood, low motivation, and suicidal thoughts, are observed in not only mood disorders, but also other psychiatric disorders, including schizophrenia and anxiety disorders (1–3). Among patients with schizophrenia, 40–50% show clinical depression (1, 2), which is known to be a risk factor for recurrence (4). Moreover, comorbid depressive symptoms are associated with clinically important issues such as reduced quality of life, increased rates of alcohol and illegal drug use, increased suicide risk, and poor adherence to medications in schizophrenia (5). Among patients with anxiety disorders, 45–95% show clinical depressive symptoms (3), which are associated with an increase in anxiety symptoms, a poor disease prognosis, and an increased risk of suicide (6, 7). Thus, depressive symptoms are considered to be clinically crucial symptoms in psychiatric disorders other than mood disorders.

Insomnia is a frequent symptom in major depressive disorder (MDD). In the Sequenced Treatment Alternatives to Relieve Depression study, a large-scale clinical trial on the treatment of MDD conducted in the USA, of 3,743 untreated outpatients with MDD, 85% had insomnia (8). Comorbid insomnia in MDD has been reported to be associated with clinically serious problems, such as poor treatment response (9, 10) and increased suicide risk (11). Based on these findings, recent studies have examined the clinical significance of interventions for comorbid insomnia in the treatment of MDD, and demonstrated that therapeutic interventions for comorbid insomnia improve not only sleep status, but also depressive symptoms (12, 13). Furthermore, previous longitudinal epidemiological studies have shown that insomnia is a major risk factor for the onset and recurrence of depression (14, 15).

Insomnia is also frequently observed in psychiatric disorders other than MDD (16). While the prevalence of insomnia in the general population is around 20% (17), it is observed in 50–80% of patients with schizophrenia (18) and in 70% of patients with anxiety disorders (19). In bipolar disorder, the prevalence ranges depending on the phase of the illness, but insomnia was seen in 81% of patients in the depressive phase in a previous study (20). As mentioned above, a strong relation between insomnia and depressive symptoms has been reported in MDD (21). In addition, this relation has been also seen in the general adult population (22, 23). Given these findings, the link between insomnia and depressive symptoms could be seen in other psychiatric disorders as well. However, to our best knowledge, no studies have examined this link in detail. If the association between insomnia and depressive symptoms is found in psychiatric disorders other than MDD, it may be possible to improve depressive symptoms *via* therapeutic interventions for insomnia in these disorders, as in MDD.

The aim of this study, which assessed sleep status both subjectively and objectively using a questionnaire and sleep electroencephalography (EEG), was to test the hypothesis that the association between insomnia and depressive symptoms can also be found in other psychiatric disorders besides MDD.

## 2. Materials and methods

### 2.1. Subjects

This study was performed using data from the SEEDs Study 2 (Practical use of sleep EEG for objective assessment and diagnosis of major depression), which aimed to construct a model of a diagnostic aid program using sleep EEG for MDD. In the SEEDs Study 2, a total of 176 in/outpatients with various psychiatric disorders were recruited, and their sleep EEG and clinical data were obtained at Shiga University of Medical Science Hospital and Nihon University Itabashi Hospital from April 2017 to March 2018.

In the present study, we used the data from 149 patients with MDD (*n* = 71), schizophrenia (*n* = 25), bipolar disorder (*n* = 27), or anxiety disorders (*n* = 26) who were enrolled in the SEEDs Study 2. As for anxiety disorders, 16 patients had generalized anxiety disorder, eight panic disorder, one social anxiety disorder, and one unspecified anxiety disorder. Diagnosis was made by trained psychiatrists according to the Diagnostic and Statistical Manual of Mental Disorders, 5th edition (DSM-5) (24) at the time of participation in the SEEDs Study 2. Because the aim of the present study was to examine the association between insomnia and depressive symptoms in psychiatric disorders, five patients with bipolar disorder were excluded because they had clinically significant manic symptoms (total score on the Young Mania Rating Scale ≥12) (25). Consequently, 144 cases were finally included in the analysis. This study was approved by the Ethics Committee of the Nihon University School of Medicine and the Shiga University of Medical Science (approval Nos. RK-210413-2 and R2021-049).

### 2.2. Measures

#### 2.2.1. Insomnia

Insomnia was assessed using the Athens Insomnia Scale (AIS) (26) for all subjects. This questionnaire consists of five items related to sleep difficulties and three related to daytime functional impairment over the past month. The score for each item ranges from 0 to 3 points, for a total of 0–24 points. Previous studies have confirmed that a score of 6 is a reasonable cutoff for insomnia (27). On this basis, an AIS score ≥6 was defined as insomnia. One MDD patient who did not respond to the AIS was excluded from the analysis. Therefore, we analyzed the AIS scores of a total of 143 patients (MDD, *n* = 70; schizophrenia, *n* = 25; bipolar disorder, *n* = 22; anxiety disorder, *n* = 26).

#### 2.2.2. Depressive symptoms

Depressive symptoms were assessed for all subjects using the Beck Depression Inventory (BDI) (28, 29). This questionnaire consists of a total of 21 questions about depressive symptoms in the last 2 weeks. The score for each item ranges from 0 to 3, for a total of 0–63 points. Patients with scores ranging from 14 to 19 are considered mildly depressed, 20 to 28 moderately depressed, and ≥29 severely depressed. Because the present study aimed to examine the association between insomnia and depressive symptoms, modified BDI (mBDI) scores were calculated by excluding the score on the sleep-related item (item No. 16, “change in sleep habits”) from the total score.

#### 2.2.3. Symptoms in schizophrenia

Clinical symptoms in patients with schizophrenia were assessed using the Positive and Negative Syndrome Scale (PANSS) (30, 31). The PANSS consists of a total of 30 items, which are assessed according to information from the past week. There are three categories: positive scale, negative scale, and general psychopathology scale. The score for each scale ranges from 7 to 49 points for the positive scale, 7–49 points for the negative scale, and 7–112 points for the general psychopathology scale, for a total score of 21–210 points.

#### 2.2.4. Manic symptoms in bipolar disorder

Manic symptoms in bipolar disorder were assessed using the Young Mania Rating Scale (25). This questionnaire consists of 11 items. The score for each item ranges from 0 to 4 or 0 to 8, for a total of 0–60 points. A score ≥12 is considered to indicate significant manic symptoms.

#### 2.2.5. Symptoms in anxiety disorders

Clinical symptoms in anxiety disorders were assessed using the State-Trait Anxiety Inventory (STAI) (32, 33). This questionnaire consists of the State Anxiety Scale (STAI-I), which assesses anxiety states, and the Trait Anxiety Scale (STAI-II), which assesses anxiety-prone personality traits. Each of the 20 items is rated on a scale of 1–4 points, with a score range of 20–80 points for both scales.

#### 2.2.6. Sleep EEG

Sleep EEG was performed using a one-channel portable electroencephalograph (Sleep Scope; SleepWell Co., Osaka, Japan). This device has been approved as a medical device in Japan (certification No. 27ADBZX00087000), and has been used in recent sleep studies (34–36). The method and analysis of the SleepScope recordings are described in detail elsewhere (37, 38). To record sleep EEG data, two electrodes were placed on the forehead and behind the auricle. The data obtained by the device were then forwarded to a cloud service (SEAS-G; SleepWell Co. Ltd.), in which the EEG data were analyzed for every 30-s epoch; the data were then classified into the following sleep stages: wake, rapid eye movement, stage N1, stage N2, and stage N3. Sleep latency was defined as 5 min of continuous sleep. Total sleep time was calculated by excluding wake time after sleep onset from the sleep period time. Sleep efficiency was defined as the ratio of total sleep time to time in bed.

Sleep EEG recording was performed from bedtime to final awakening on 2 consecutive nights. To avoid the “first night effect” (39, 40), the data from the second night were used for analysis. However, if the recordings on the second night failed (e.g., interruption of recording due to dead battery or electrode dislocation), the recordings from the first night were used. Cases where valid data could not be obtained on both nights included one case of MDD, one case of schizophrenia, and one case of bipolar disorder. Therefore, we analyzed EEG data from a total of 141 patients (MDD, *n* = 70; schizophrenia, *n* = 24; bipolar disorder, *n* = 21; anxiety disorder, *n* = 26). According to previous studies (41, 42), insomnia was defined by sleep latency ≥31 min and/or wake time after sleep onset ≥31 min.

### 2.3. Statistical analysis

Demographic data (age and sex) and medication doses were compared between the insomnia and non-insomnia groups. The average daily dosage was expressed as chlorpromazine for antipsychotics, imipramine for antidepressants, and diazepam for benzodiazepines (43). In all patients with bipolar disorder, mood stabilizers were used (lithium, *n* = 10; valproate, *n* = 8; lamotrigine, *n* = 3; carbamazepine, *n* = 2). The insomnia and non-insomnia groups were defined using the AIS and sleep EEG, separately. Sex was compared using the chi-square test, and other continuous variables were compared using a *t*-test. *T*-tests were also used for comparing the scores for depressive symptoms (mBDI score), but when any of the demographic data or medication doses had a significant difference between groups, analysis of covariance was performed with these factors as covariates. Correlations between the mBDI score and sleep parameters obtained using sleep EEG were examined by Pearson’s correlation analysis. In order to confirm the clinical significance of depressive symptoms in schizophrenia and anxiety disorders, we assessed the association between the score for depressive symptoms (mBDI score) and the score for clinical symptoms specific to these disorders (PANSS and STAI scores) by Pearson’s correlation analysis. Statistical analysis was performed using SPSS version 25 (IBM Corp., Armonk, NY, USA), and was considered significant when *p* < 0.05.

## 3. Results

### 3.1. Comparisons of demographic data and medication dosage between the insomnia and non-insomnia groups as defined by AIS scores

Table 1 shows the demographic data and daily medication dosage in the insomnia and non-insomnia groups as defined by AIS scores. The prevalence of insomnia was 77.1% for MDD, 36.0% for schizophrenia, 63.6% for bipolar disorder, and 69.2% for anxiety disorder. In MDD, antidepressant doses were higher in patients without insomnia than in those with insomnia (*t* = 3.66, *p* = 0.001). In bipolar disorder, age was higher in patients without insomnia than in those with insomnia (*t* = 3.59, *p* = 0.002).

**TABLE 1:** Comparison of background information between the insomnia and non-insomnia groups as defined by Athens insomnia scale scores.

	Whole sample	Insomnia	Non-insomnia	Unadjusted		Adjusted*	
	**Mean (SD) or *n***			**χ^2^ or *t***	***p***	**F**	***p***
**MDD**	*n* = 70	*n* = 54	*n* = 16				
Age	45.5 (13.3)	45.2 (13.0)	46.3 (14.6)	0.27^b^	n.s.		
Sex (M/F)	22/48	14/40	8/8	3.32^a^	n.s.		
Medication							
CP (mg/day)	52.0 (139.4)	24.5 (67.0)	144.7 (248.4)	1.92^b^	n.s.		
IMIP (mg/day)	122.8 (101.8)	100.5 (86.2)	198.0 (116.6)	3.66^b^	0.001		
DZP (mg/day)	8.4 (19.8)	9.1 (22.0)	5.9 (8.8)	−0.57^b^	n.s.		
mBDI	22.5 (11.5)	25.6 (10.7)	12.1 (6.9)	−4.71^b^	<0.001	15.85*^c^*	<0.001
**Schizophrenia**	*n* = 25	*n* = 9	*n* = 16				
Age	42.8 (9.9)	44.8 (6.0)	41.6 (11.6)	−0.76^b^	n.s.		
Sex (M/F)	7/18	2/7	5/11	0.23^a^	n.s.		
Medication							
CP (mg/day)	523.0 (335.6)	499.8 (318.7)	536.0 (354.3)	0.25^b^	n.s.		
IMIP (mg/day)	9.50 (32.9)	16.7 (50.0)	5.5 (18.8)	−0.81^b^	n.s.		
DZP (mg/day)	10.8 (15.0)	12.4 (15.3)	9.9 (15.3)	−0.38^b^	n.s.		
mBDI	15.3 (9.4)	22.8 (8.6)	11.1 (7.0)	−3.68^b^	0.001		
PANSS	49.8 (19.5)	65.3 (14.2)	41.6 (16.9)	−3.37^b^	0.003		
**Bipolar disorder**	*n* = 22	*n* = 14	*n* = 8				
Age	44.7 (12.6)	38.9 (11.2)	54.9 (7.5)	3.59^b^	0.002		
Sex (M/F)	5/17	3/11	2/6	0.037^a^	n.s.		
Medication							
CP (mg/day)	235.7 (384.5)	203.9 (310.8)	291.4 (508.5)	0.50^b^	n.s.		
IMIP (mg/day)	51.0 (91.8)	30.4 (53.0)	87.0 (133.1)	1.15^b^	n.s.		
DZP (mg/day)	17.4 (20.9)	17.9 (23.4)	16.6 (17.2)	−0.13^b^	n.s.		
mBDI	23.5 (11.0)	28.6 (9.5)	14.5 (7.4)	−3.61^b^	0.002	8.34*^c^*	0.009
YMRS	3.8 (3.5)	4.3 (3.6)	2.9 (3.3)	−0.91^b^	n.s.	−0.91*^c^*	n.s.
**Anxiety disorders**	*n* = 26	*n* = 18	*n* = 8				
Age	42.3 (14.7)	42.4 (14.6)	42.0 (16.1)	−0.070^b^	n.s.		
Sex (M/F)	8/18	6/12	2/6	0.18^a^	n.s.		
Medication							
CP (mg/day)	8.0 (30.1)	1.1 (4.5)	23.5 (52.8)	1.20^b^	n.s.		
IMIP (mg/day)	52.4 (68.8)	46.5 (75.7)	65.6 (52.1)	0.65^b^	n.s.		
DZP (mg/day)	10.1 (21.7)	13.8 (25.3)	1.8 (2.9)	−1.32^b^	n.s.		
mBDI	20.4 (11.1)	23.9 (10.4)	12.5 (8.8)	−2.70^b^	0.012		
STAI-I	49.3 (8.5)	51.3 (8.9)	44.8 (5.7)	−1.90^b^	n.s.		
STAI-II	57.1 (9.1)	59.8 (8.3)	51.0 (8.4)	−2.50^b^	0.019		

MDD, major depressive disorder; CP, chlorpromazine; IMIP, imipramine; DZP, diazepam; mBDI, modified beck depression inventory; PANSS, positive and negative syndrome scale; YMRS, young mania rating scale; STAI-I, state anxiety scale; STAI-II, trait anxiety scale.

*When any of the demographic data (age and sex) or medication doses had a significant difference between groups, analysis of covariance was performed with these factors as covariates.

^a^Chi-square test.

^b^*t*-test.

^c^Analysis of covariance.

### 3.2. Comparison of demographic data and medication dosage between the insomnia and non-insomnia groups as defined by sleep EEG

Table 2 shows the demographic data and daily medication dosage in the insomnia and non-insomnia groups as defined by sleep EEG. The prevalence of insomnia was 82.9% for MDD, 70.8% for schizophrenia, 81.0% for bipolar disorder, and 69.2% for anxiety disorder. In MDD, antidepressant doses were higher in patients with insomnia than in those without insomnia (*t* = −2.05, *p* = 0.044). In bipolar disorder, antipsychotic doses were higher in patients with insomnia than in those without insomnia (*t* = −2.11, *p* = 0.048).

**TABLE 2:** Comparison of background information between the insomnia and non-insomnia groups as defined by sleep electroencephalography.

	Whole sample	Insomnia	Non-insomnia	Unadjusted		Adjusted*	
	**Mean (SD) or *n***			**χ^2^ or *t***	***p***	**F**	***p***
**MDD**	*n* = 70	*n* = 58	*n* = 12				
Age	45.8 (13.6)	45.0 (13.9)	49.9 (11.8)	1.15^b^	n.s.		
Sex (M/F)	23/47	18/40	5/7	0.51^a^	n.s.		
Medication							
CP (mg/day)	52.0 (139.4)	51.9 (133.1)	52.3 (173.6)	0.010^b^	n.s.		
IMIP (mg/day)	119.7 (98.8)	130.5 (100.6)	67.7 (71.7)	−2.05^b^	0.044		
DZP (mg/day)	8.3 (19.8)	6.3 (10.4)	17.8 (42.1)	0.94^b^	n.s.		
mBDI	22.6 (11.3)	22.5 (11.1)	23.1 (13.0)	0.15^b^	n.s.	0.12*^c^*	n.s.
**Schizophrenia**	*n* = 24	*n* = 17	*n* = 7				
Age	41.9 (9.2)	43.3 (7.7)	38.6 (12.0)	−1.16^b^	n.s.		
Sex (M/F)	6/18	4/13	2/5	0.067^a^	n.s.		
Medication							
CP (mg/day)	523.9 (342.8)	533.6 (310.3)	500.3 (439.0)	−0.21^b^	n.s.		
IMIP (mg/day)	9.9 (33.6)	4.4 (18.2)	23.2 (56.1)	0.87^b^	n.s.		
DZP (mg/day)	10.7 (15.3)	9.3 (13.0)	13.9 (9.4)	0.65^b^	n.s.		
mBDI	15.7 (9.4)	18.1 (9.3)	9.9 (7.1)	−2.11^b^	0.047		
PANSS	49.0 (19.5)	53.6 (18.5)	36.8 (17.9)	−1.90^b^	n.s.		
**Bipolar disorder**	*n* = 21	*n* = 17	*n* = 4				
Age	45.2 (12.6)	45.8 (11.5)	43.0 (18.8)	−0.39^b^	n.s.		
Sex (M/F)	5/16	5/12	0/4	1.54^a^	n.s.		
Medication							
CP (mg/day)	247.0 (390.3)	290.4 (423.2)	62.5 (66.1)	−2.11^b^	0.048		
IMIP (mg/day)	53.4 (93.3)	62.6 (101.3)	14.1 (28.1)	−0.93^b^	n.s.		
DZP (mg/day)	17.6 (21.4)	18.5 (22.2)	13.8 (20.5)	−0.39^b^	n.s.		
mBDI	22.6 (10.6)	23.1 (10.6)	20.8 (11.6)	−0.39^b^	n.s.		
YMRS	3.7 (3.5)	3.8 (3.9)	3.3 (1.5)	−0.43^b^	n.s.	0.05*^c^*	n.s.
**Anxiety disorders**	*n* = 26	*n* = 18	*n* = 8				
Age	42.3 (14.7)	43.1 (15.0)	40.6 (15.1)	−0.38^b^	n.s.		
Sex (M/F)	8/18	5/13	3/5	0.25^a^	n.s.		
Medication							
CP (mg/day)	8.0 (30.1)	11.5 (35.9)	0	−0.90^b^	n.s.		
IMIP (mg/day)	52.4 (68.8)	62.5 (78.4)	29.7 (34.0)	−1.13^b^	n.s.		
DZP (mg/day)	10.1 (21.7)	6.2 (7.5)	18.9 (37.6)	0.94^b^	n.s.		
mBDI	20.4 (11.1)	22.3 (10.9)	16.0 (10.8)	−1.37^b^	n.s.		
STAI-I	49.3 (8.5)	50.2 (9.3)	47.3 (6.5)	−0.80^b^	n.s.		
STAI-II	57.1 (9.1)	58.0 (10.0)	55.1 (6.9)	−0.73^b^	n.s.		

MDD, major depressive disorder; CP, chlorpromazine; IMIP, imipramine; DZP, diazepam; mBDI, modified beck depression inventory; PANSS, positive and negative syndrome scale; YMRS, young mania rating scale; STAI-I, state anxiety scale; STAI-II, trait anxiety scale.

*When any of the demographic data (age and sex) or medication doses had a significant difference between groups, analysis of covariance was performed with these factors as covariates.

^a^Chi-square test.

^b^*t*-test.

^c^Analysis of covariance.

### 3.3. Comparison of depressive symptoms between the insomnia and non-insomnia groups as defined by AIS scores

**FIGURE 1:**
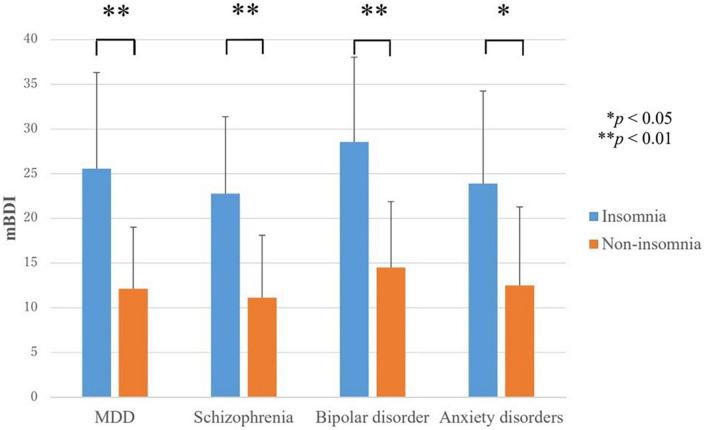
Severity of depressive symptoms in the insomnia and non-insomnia groups as defined by Athens insomnia scale scores. MDD, major depressive disorder; mBDI, modified beck depression inventory. In all disorders, the score for depressive symptoms (mBDI) were higher in the insomnia group than in the non-insomnia group. *P < 0.05, ^**^P < 0.01.

In all disorders, the score for depressive symptoms (mBDI) were higher in the insomnia group than in the non-insomnia group (Figure 1). In MDD, the mBDI scores for the insomnia group and non-insomnia group were 25.6 ± 10.7 and 12.1 ± 6.9, respectively (*F* = 15.9, *p* < 0.001). In schizophrenia, the mBDI scores for the insomnia group and non-insomnia group were 22.8 ± 8.6 and 11.1 ± 7.0, respectively (*t* = −3.7, *p* = 0.001). In bipolar disorder, the mBDI scores for the insomnia group and non-insomnia group were 28.6 ± 9.5 and 14.5 ± 7.4, respectively (*F* = 8.3, *p* = 0.009). In anxiety disorders, the mBDI scores for the insomnia group and non-insomnia group were 23.9 ± 10.4 and 12.5 ± 8.8, respectively (*t* = −2.7, *p* = 0.012).

### 3.4. Comparisons of depressive symptoms between the insomnia and non-insomnia groups as defined by sleep EEG

**FIGURE 2:**
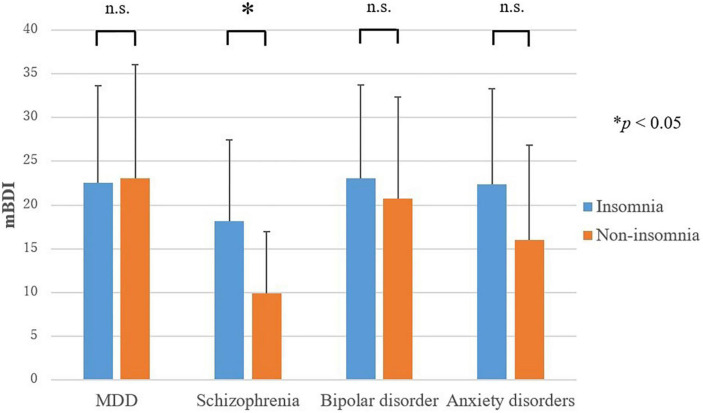
Severity of depressive symptoms in the insomnia and non-insomnia groups as defined by sleep electroencephalography. MDD, major depressive disorder; mBDI, modified beck depression inventory. Only in schizophrenia, the score for depressive symptoms (mBDI) were higher in the insomnia group than in the non-insomnia group. *P < 0.05.

Only in schizophrenia, a significant difference was found for the score for depressive symptoms (mBDI) between the insomnia group and non-insomnia group; depressive symptoms were more severe in the insomnia group than in the non-insomnia group (*t* = −2.1, *p* = 0.047) (Figure 2). The mBDI scores for the insomnia group and non-insomnia group were 18.1 ± 9.3 and 9.9 ± 7.1, respectively. In MDD, bipolar disorder, and anxiety disorders, no difference was found in the score for depressive symptoms between the insomnia group and non-insomnia group.

### 3.5. Associations between depressive symptoms and sleep parameters obtained with sleep EEG (Supplementary material)

In bipolar disorder, total sleep time was negatively correlated with mBDI scores (*r* = −0.47, *p* = 0.03); however, no other correlations with mBDI scores were found for the other sleep parameters. In MDD, schizophrenia, and anxiety disorders, no sleep parameters showed significant associations with mBDI scores. However, in schizophrenia, time in bed (*r* = 0.36, *p* = 0.09), wake time after sleep onset (*r* = 0.38, *p* = 0.07), and sleep latency (*r* = 0.37, *p* = 0.08) showed positive correlation trends.

### 3.6. Associations between depressive symptoms and disorder-specific symptoms in schizophrenia and anxiety disorders

In schizophrenia, a positive correlation was found between mBDI scores and PANSS total scores (*r* = 0.52, *p* = 0.011), implying that the more severe the depressive symptoms, the more severe the schizophrenia symptoms. Positive correlations with the mBDI scores were also found for all three PANSS subscales: positive symptom scale (*r* = 0.74, *p* < 0.001), negative symptom scale (*r* = 0.61, *p* = 0.002), and general psychopathology scale (*r* = 0.81, *p* < 0.001).

In anxiety disorders, a positive correlation was found between mBDI scores and STAI-I scores (*r* = 0.63, *p* = 0.001), implying that the more severe the depressive symptoms, the more severe the anxiety symptoms. Similarly, a positive correlation was found between mBDI scores and STAI-II scores (*r* = 0.81, *p* < 0.001).

## 4. Discussion

The present study examined the association between sleep status and depressive symptoms in major psychiatric disorders, including MDD, bipolar disorder, schizophrenia, and anxiety disorders, and found that subjective insomnia, as defined by the AIS, was associated with depressive symptoms in all disorders. In other words, this association was transdiagnostic in major psychiatric disorders. Furthermore, in schizophrenia, a relation between depressive symptoms and insomnia was also found by objective sleep assessment methods using sleep EEG. To our best knowledge, this is the first study to evaluate the association between sleep status and depressive symptoms in multiple psychiatric disorders in the same manner.

Depressive symptoms have been considered clinically significant in psychiatric disorders because they relate to important clinical issues such as reduced quality of life and increased risk of suicide (4–7). Moreover, in the present study, we confirmed that depressive symptoms correlate with the core symptoms of schizophrenia and anxiety disorders. Based on these findings, focusing on depressive symptoms could be crucial in improving the prognosis of major psychiatric disorders.

In MDD, therapeutic interventions for comorbid insomnia, such as the addition of hypnotics or cognitive behavioral therapy for insomnia to antidepressant treatment, are effective for improving not only insomnia, but also depressive symptoms (12, 13). Given the present finding that the association between insomnia and depressive symptoms was transdiagnostic in major psychiatric disorders, these interventions for comorbid insomnia could also be effective for improving depressive symptoms in psychiatric disorders other than MDD. In addition, it may also be useful to select an agent having more sedative properties among the therapeutic drugs for each disorder (e.g., olanzapine or asenapine for schizophrenia). Further research is needed to clarify this point in order to establish treatment strategies to improve the prognosis of major psychiatric disorders.

The prevalence of subjective insomnia as defined by the AIS was highest in depression (77.1%), but was similar in anxiety disorders (69.2%) and bipolar disorder (63.6%). These results were comparable to previous studies (8, 19, 20). Compared with these disorders, the prevalence of subjective insomnia (36.0%) in schizophrenia was lower; however, that of objective insomnia as defined by sleep EEG (70.8%) was similar to those of the other disorders. This large discrepancy of the prevalence between subjective and objective insomnia in schizophrenia may be explained by a cognitive characteristic of this disorder. Bian et al. (44) reported that patients with schizophrenia, especially in the chronic phase, tended to overestimate their sleep time, and that 38.5% have such misperception of sleep time.

In MDD, bipolar disorder, and anxiety disorders, no association was found between depressive symptoms and objective insomnia as defined by sleep EEG. These results differed from those of subjective insomnia assessed using the AIS. Patients with depression, in the depressive phase of bipolar disorder, and with anxiety disorders are known to show a tendency to perceive their own symptoms to be worse than they actually are (45–49). This pessimistic perception may have led to an overestimation of insomnia symptoms compared with objective findings. In these disorders, subjective sleep perception could be useful to detect poor sleep status in relation to depressive symptoms rather than objective sleep assessment methods. In schizophrenia, the relation of depressive symptoms with insomnia was found by objective sleep assessment methods using sleep EEG. Therefore, in addition to subjective sleep perception, sleep EEG indices could be useful to detect poor sleep status in relation to depressive symptoms in schizophrenia. This result may be explained by the fact that they are less likely to overestimate their insomnia (44). These results suggest that sleep EEG could also be useful to detect poor sleep status related to depressive symptoms in schizophrenia.

In the correlation analyses between sleep EEG parameters and depressive symptoms, sleep latency and wake time after sleep onset showed positive correlation trends in schizophrenia, reflecting the results of the comparisons of depressive symptom severity between the insomnia and non-insomnia groups as defined by sleep EEG. In bipolar disorder, total sleep time was negatively associated with depressive symptoms, but this association was not found in MDD, schizophrenia, or anxiety disorders. Because the sample size in the present study was small, whether this finding is specific to bipolar disorder needs to be investigated in larger samples.

### 4.1. Study limitations

Our results should be viewed in light of some methodological limitations. First, the present study did not include patients who were unable to complete the questionnaire or who were unable to undergo sleep EEG. Therefore, it may be that the present findings cannot be generalized for severe cases. Second, the sample size was small, so it is possible that significant differences were not found for some study items because of the low statistical power. In addition, the number of patients with depression was larger than the number of patients with other psychiatric disorders, which also may have affected the results. In the future, it needs to be confirmed whether similar results can be obtained with a larger sample size and without disease bias. Third, although age, sex, and medication were adjusted in the comparisons between the insomnia and non-insomnia groups, other potential factors, such as alcohol consumption, may have influenced the outcome. Finally, the present study was a cross-sectional study; therefore, it is not possible to assess causal relationships between insomnia and depressive symptoms. Further longitudinal studies need to clarify these causal relationships.

## 5. Conclusion

The association between subjective insomnia and depressive symptoms was a transdiagnostic feature in major psychiatric disorders. Further studies are needed to clarify whether therapeutic interventions for comorbid insomnia can improve depressive symptoms in major psychiatric disorders, similar to MDD.

## Data availability statement

The raw data supporting the conclusions of this article will be made available by the authors, without undue reservation.

## Ethics statement

The studies involving human participants were reviewed and approved by the Ethics Committee of the Nihon University School of Medicine and the Shiga University of Medical Science. The patients/participants provided their written informed consent to participate in this study.

## Author contributions

SN, YK, and MS conceived the study. SN performed the statistical analysis and drafted the manuscript under supervision of YK and MS. SN, YK, NF, JK, KS, and MS interpreted the data. NF, JK, KS, KN, AK, TY, HK, NY, MU, and KK contributed to the revision of the draft manuscript. All authors contributed to the data collection and approved the final version of the manuscript.
