# Supplementary material for: Transdiagnostic association between subjective insomnia and depressive symptoms in major psychiatric disorders
Source: Front Psychiatry. 2023 Apr 24;14:1114945. doi: 10.3389/fpsyt.2023.1114945 (PMC10165079; doi:10.3389/fpsyt.2023.1114945)
Supplement: Supplementary file 1 [file Table_1.DOCX]

Transdiagnostic association between subjective insomnia and depressive symptoms in major psychiatric disorders

Suguru Nakajima^1^, Yoshiyuki Kaneko^1*^, Nobukuni Fujii^1^, Jun Kizuki^1, 2^, Kaori Saitoh^1^, Kentaro Nagao^2^, Aoi Kawamura^2^, Takuya Yoshiike^2^, Hiroshi Kadotani^3^, Naoto Yamada^4^, Makoto Uchiyama^1, 5^ Kenichi Kuriyama^2^, Masahiro Suzuki^1*^

^1^ Department of Psychiatry, Nihon University School of Medicine, Tokyo 173-8610, Japan

^2^ Department of Sleep-Wake Disorders, National Institute of Mental Health, National Center of Neurology and Psychiatry, Tokyo, Japan

^3^ Department of Psychiatry, Shiga University of Medical Science, Shiga, Japan

^4^ Kamibayashi Memorial Hospital, Aichi, Japan

^5^ Tokyo Adachi Hospital, Tokyo, Japan

*** Correspondence:**Masahiro Suzuki
[suzuki.masahiro94@nihon-u.ac.jp](about:blank)

Yoshiyuki Kaneko
[kaneko.yoshiyuki@nihon-u.ac.jp](about:blank)

# Supplementary Table

**Correlations between mBDI and sleep parameters obtained with sleep electroencephalography**

|  | **MDD** | **Schizophrenia** | **Bipolar disorder** | **Anxiety disorders** |
| --- | --- | --- | --- | --- |
|  | **mBDI** | | | |
| Time in bed | r = -0.11  p = 0.376 | r = 0.36  p = 0.086 | r = -0.19  p = 0.401 | r = 0.08  p = 0.684 |
| Total sleep time | r = 0.03  p = 0.778 | r = 0.12  p = 0.593 | r = -0.47  p = 0.031* | r = 0.11  p = 0.591 |
| Wake time after sleep onset | r = -0.19  p = 0.116 | r = 0.38  p = 0.067 | r = 0.00  p = 0.999 | r = -0.18  p = 0.383 |
| Sleep efficiency | r = 0.15  p = 0.217 | r = -0.33  p = 0.113 | r = -0.23  p = 0.321 | r = -0.01  p = 0.967 |
| Sleep latency | r = -0.15  p = 0.206 | r = 0.37  p = 0.075 | r = -0.14  p = 0.547 | r = 0.27  p = 0.181 |
| REM latency | r = 0.04  p = 0.764 | r = 0.09  p = 0.670 | r = -0.25  p = 0.269 | r = -0.12  p = 0.569 |
| Stage Wake | r = -0.16  p = 0.196 | r = 0.29  p = 0.166 | r = 0.19  p = 0.414 | r = -0.15  p = 0.476 |
| Stage N1 | r = 0.05  p = 0.685 | r = 0.06  p = 0.780 | r = -0.21  p = 0.374 | r = -0.29  p = 0.155 |
| Stage N2 | r = 0.15  r = 0.219 | r = -0.23  p = 0.291 | r = 0.12  p = 0.597 | r = 0.20  p = 0.338 |
| Stage N3 | r = 0.02  p = 0.903 | r = 0.05  p = 0.827 | r = -0.14  p = 0.533 | r = -0.03  p = 0.886 |
| Stage REM | r = 0.01  p = 0.916 | r = -0.47  p = 0.828 | r = -0.34  p = 0.129 | r = 0.09  p = 0.680 |

*p<0.05

mBDI: modified Beck Depression Inventory, MDD: major depressive disorder,

REM: Rapid Eye Movement
